# Pancreatic cancer circulating tumour cells express a cell motility gene signature that predicts survival after surgery

**DOI:** 10.1186/1471-2407-12-527

**Published:** 2012-11-16

**Authors:** Gregory Sergeant, Rudy van Eijsden, Tania Roskams, Victor Van Duppen, Baki Topal

**Affiliations:** 1 Department of Abdominal Surgery, University Hospitals Leuven, Herestraat 49, Leuven, 3000, Belgium; 2 VIB Nucleomics Core, KU Leuven, Herestraat 49, Leuven, 3000, Belgium; 3 Department of Pathology, University Hospitals Leuven, Herestraat 49, Leuven, 3000, Belgium; 4 Laboratory of Experimental Hematology, KU Leuven, Herestraat 49, Leuven, 3000, Belgium

**Keywords:** Circulating tumour cells, Pancreatic ductal adenocarcinoma, Gene expression profiling, p38 – MAPK signaling, Transforming growth factor - β1, Cancer cell migration, Cell motility

## Abstract

**Background:**

Most cancer deaths are caused by metastases, resulting from circulating tumor cells (CTC) that detach from the primary cancer and survive in distant organs. The aim of the present study was to develop a CTC gene signature and to assess its prognostic relevance after surgery for pancreatic ductal adenocarcinoma (PDAC).

**Methods:**

Negative depletion fluorescence activated cell sorting (FACS) was developed and validated with spiking experiments using cancer cell lines in whole human blood samples. This FACS-based method was used to enrich for CTC from the blood of 10 patients who underwent surgery for PDAC. Total RNA was isolated from 4 subgroup samples, i.e. CTC, haematological cells (G), original tumour (T), and non-tumoural pancreatic control tissue (P). After RNA quality control, samples of 6 patients were eligible for further analysis. Whole genome microarray analysis was performed after double linear amplification of RNA. ‘Ingenuity Pathway Analysis’ software and AmiGO were used for functional data analyses. A CTC gene signature was developed and validated with the nCounter system on expression data of 78 primary PDAC using Cox regression analysis for disease-free (DFS) and overall survival (OS).

**Results:**

Using stringent statistical analysis, we retained 8,152 genes to compare expression profiles of CTC vs. other subgroups, and found 1,059 genes to be differentially expressed. The pathway with the highest expression ratio in CTC was p38 mitogen-activated protein kinase (p38 MAPK) signaling, known to be involved in cancer cell migration. In the p38 MAPK pathway, TGF-β1, cPLA2, and MAX were significantly upregulated. In addition, 9 other genes associated with both p38 MAPK signaling and cell motility were overexpressed in CTC. High co-expression of TGF-β1 and our cell motility panel (≥ 4 out of 9 genes for DFS and ≥ 6 out of 9 genes for OS) in primary PDAC was identified as an independent predictor of DFS (p=0.041, HR (95% CI) = 1.885 (1.025 – 3.559)) and OS (p=0.047, HR (95% CI) = 1.366 (1.004 – 1.861)).

**Conclusions:**

Pancreatic CTC isolated from blood samples using FACS-based negative depletion, express a cell motility gene signature. Expression of this newly defined cell motility gene signature in the primary tumour can predict survival of patients undergoing surgical resection for pancreatic cancer.

**Trial Registration:**

Clinical trials.gov NCT00495924

## Background

The vast majority of patients suffering from solid organ malignancies ultimately die from metastases, irrespective of the type of treatment modality [1]. Current cancer treatments are mainly based on primary tumour characterization instead of on characterization of metastases or cancer cells in the blood circulation. Since distant organ metastases are the end-results of circulating tumor cells (CTC), molecular characterization of CTC is promising in the quest for effective therapeutic agents and novel prognostic markers [2].

Unfortunately, specific isolation of CTC has proved difficult. Identification and isolation of CTC using predefined marker expression has failed to identify certain cancer cell phenotypes, and consequently also their genotypes. Methods to isolate, identify and enable reliable and reproducible molecular characterization of CTC without a priori selection could provide new insights into cancer research and improve management of the disease.

The aim of the present study was to develop a negative depletion strategy to enrich whole blood for CTC without excluding any particular subset of CTC, and to study their gene-expression profiles in order to find novel therapeutic targets and prognostic markers in patients with pancreatic cancer.

## Methods

### Patients and tissues

Fresh tumour (T) and surrounding non-tumoural control (P) tissue samples were obtained from surgical specimens from 10 patients (M/F: 5/5; median age (range): 61,0 (50 – 74) years) who had undergone surgical resection for pancreatic ductal adenocarcinoma (PDAC) (Table 1). To verify that samples contained more than 70% tumour tissue, hematoxylin and eosin stains were made from each tumour sample and from surrounding control pancreatic tissue to confirm its non-cancerous histology. Tissue samples were immediately submerged in RNAlater® (Qiagen) and stored at −80°C. From the same 10 patients, 20ml of EDTA-treated venous blood was drawn intra-operatively via a central venous line, immediately after removal of the resection specimen. Patient recruitment started after approval of the study by the ethical committee of the University Hospital Leuven (Ref. ML 3452) and complete registration of the protocol at Clinicaltrials.gov [NCT00495924]. The research reported was undertaken in compliance with the Helsinki Declaration. Informed consent was obtained from all patients included.

**Table 1:** Clinicopathological characteristics of included patients

**Code**	**Gender**	**Age (years)**	**Histology**	**Tumor grade**	**T-stage**	**N-stage**	**AJCC classif. (2002)**
77	Female	63	PDAC	3	3	1	2b
87	Male	57	PDAC	1	3	1	2b
88	Female	48	PDAC	2	3	1	2b
90	Male	59	PDAC	1	3	0	2a
91*	Female	51	PDAC	3	3	0	2a
104*	Female	72	PDAC	3	3	1	2b
105*	Male	68	PDAC	3	2	0	1b
117*	Female	72	PDAC	3	3	0	2a
123*	Male	74	PDAC	3	1	1	2b
138*	Male	50	PDAC	2	2	1	2b

PDAC: pancreatic ductal adenocarcinoma. * indicates patients of whom all samples reached RNA quality standards set for microarray analysis.

### Fluorescence activated cell sorting (FACS)

**Figure 1:**
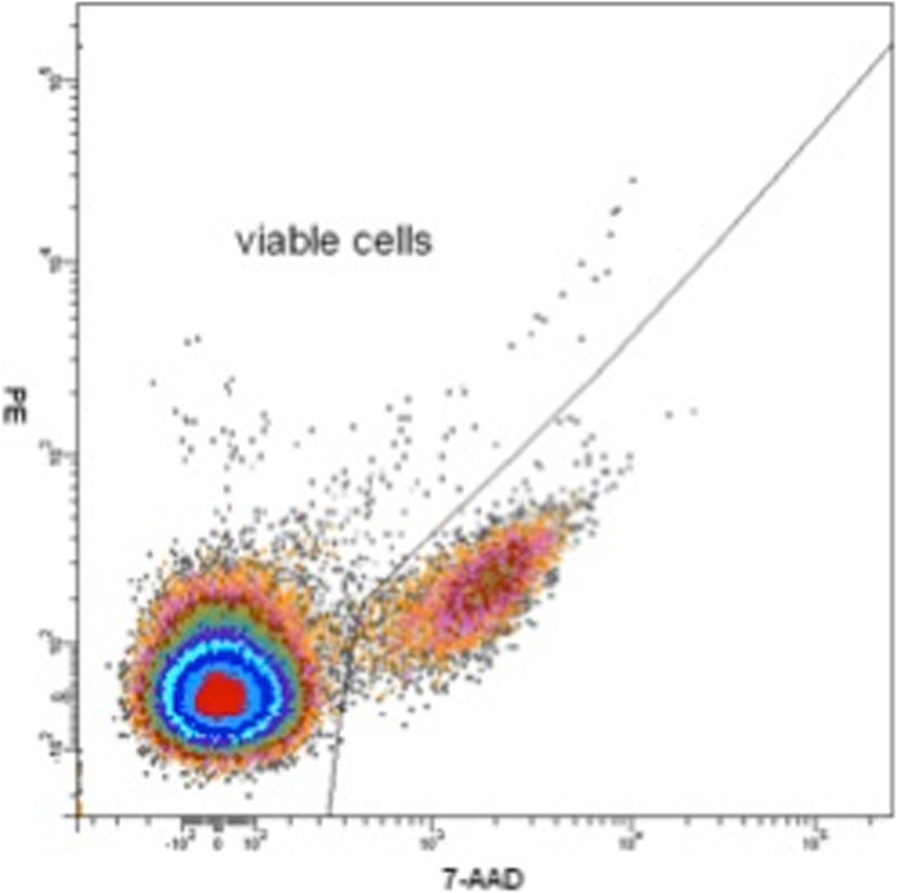
Density plot showing how 7-AAD positive non-viable cells were excluded from viable cells.

**Figure 2:**
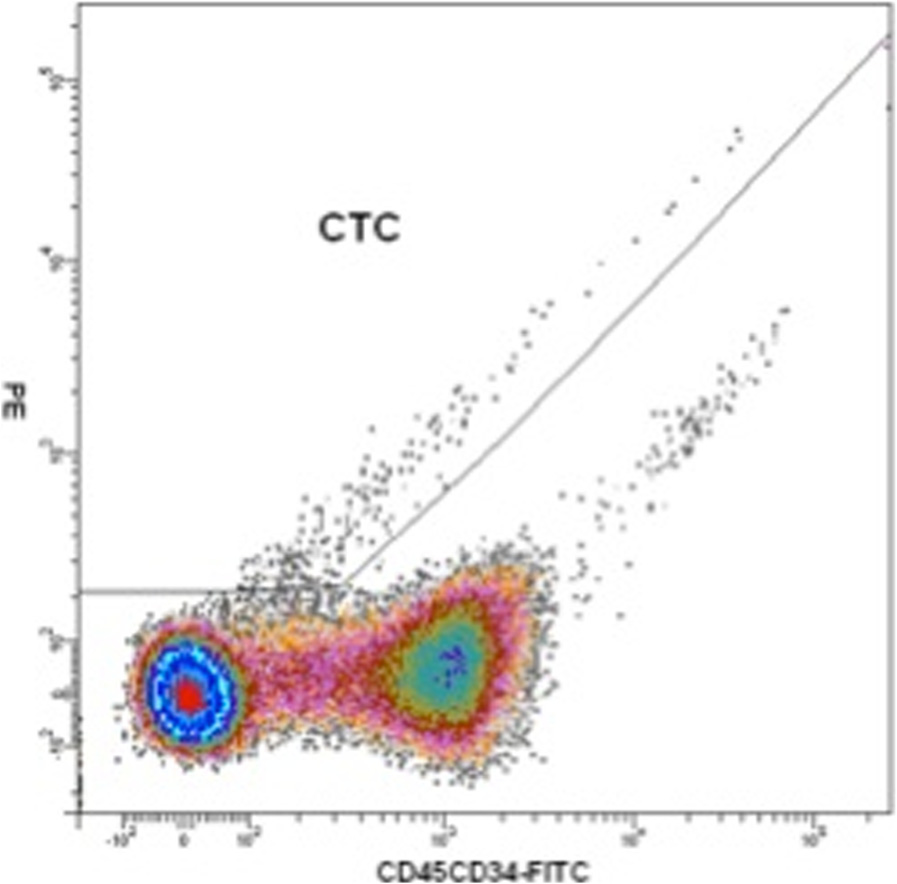
**Density plot presenting viable cells and exclusion of CD45CD34-FITC positive and negative cells.** CTC population is a subpopulation of viable cells. CTC: circulating tumour cells.

Blood samples were incubated on ice in Oncoquick® tubes (Greiner Bio-One, GmbH, Kremsmünster, Austria) for 10–15 minutes [3,4]. Enrichment of tumour cells was obtained by density gradient centrifugation following the manufacturer’s recommendations at a centrifugation acceleration of 1400g. The interphase was collected and washed twice with washing buffer. The cell pellet was then resuspended in 400ml phosphate buffered saline (PBS) and transferred to a polystyrene FACS-tube. Subsequently, the cells were stained by incubating at 4°C with 20μl of directly conjugated anti-CD45 and anti-CD34 FITC-labelled antibodies (BD Biosciences) for 30 minutes. The sample was washed and resuspended in 1ml of PBS. Non-viable cells were stained by incubation with 10μl of the 7-amino-actinomycin D (Calbiochem, Merck Chemical Ltd, Nottingham, UK) for 15–30 minutes. The analysis was done on a FACSDiva™ 6.0 upgraded FACSVantage™ SE cell sorter (BD Biosciences). A negative depletion procedure was used to enrich for CTC, combining gates containing CD45 negative and CD34 negative cells in addition to 7-amino-actinomycin D viability staining to exclude all haematological and non-viable (G fraction) cells (Figures 1 and 2). Photomultiplicator (PMT) settings were chosen based on fluorophore-labelled beads. For gene expression analysis all fractions were directly sorted in RLT (Qiagen) on ice.

### Spiking experiments

The Panc-1 pancreatic cancer cell line was purchased from the American Type Culture Collection (ATCC; CRL-1469, ABPromochem) [5]. Panc-1 cells were cultured at 37°C in DMEM supplemented with 10% foetal bovine serum, penicillin and streptomycin.

Two different concentrations of Panc-1 cells (5.0 × 10^4^ and 5.0 × 10^6^ cells) were spiked in duplo in 20ml of EDTA-treated blood obtained from healthy volunteers, and gently versed in an Oncoquick® tube. The recovered cell populations (CTC- and G-fractions) were FACS-sorted in CytoRich® red and cytospins were made by spinning the cells at 800 rpm for 10min. Slides were air-dried for 24 hours and a pre-keratin stain (anti-CK8/18, 1:30, Novacastra) was carried out. Cells were counterstained with haematoxylin. An experienced pathologist (TR) semi-quantitatively scored the relative distribution of cell populations.

### RNA isolation

RNA was isolated from tissue sections using a protocol combining Trizol/chloroform extraction, followed by column chromatography with the RNeasy Mini kit (Qiagen). Briefly, after homogenization of the tissue with 500μl of Trizol® (Invitrogen), 100μl of chloroform was added followed by mixing until a milky solution was obtained.

This mixture was then centrifuged at 12.000 rpm in a cooled benchtop centrifuge for 25 minutes. Subsequently, the supernatant was removed and added to an equal amount of 70% ethanol. After this step the RNeasy Mini kit was followed according to the manufacturer’s recommendations.

RNA from the FACS sorted cell populations was prepared using the RNeasy Micro kit (Qiagen), including a DNase step according to the manufacturer’s recommendations.

### Array hybridization

RNA concentration and purity were determined spectrophotometrically using the Nanodrop ND-1000 (Nanodrop Technologies) for all samples. RNA integrity of the T and P samples was assessed using a Bioanalyser 2100 (Agilent). Per sample, ideally an amount of 5–10 ng of total RNA spiked with four bacterial RNA transcripts (Affymetrix) was converted and amplified to double-stranded cDNA in a 2-cycle cDNA reverse transcription reaction. Subsequently, the sample was converted to antisense cRNA and labelled with biotin through an *in vitro* transcription reaction according to the manufacturer’s protocol (Affymetrix). Purified fragmented biotinylated cRNA and hybridization controls (Affymetrix) were mixed, hybridized on Affymetrix HG U133 Plus 2.0 arrays, and subsequently stained and washed in the GeneChip fluidics station 450 (Affymetrix). To assess the raw probe signal intensities, chips were scanned using the GeneChip scanner 3000 (Affymetrix).

### Microarray data analysis

R, a free software environment for statistical computing and graphics, was used in combination with the *affy* and *limma* libraries in Bioconductor for microarray data analysis [6-9]. To decide whether a signal was significantly above background, the MAS 5.0 algorithm was applied to calculate probe set detection calls. Robust Multichip Average (RMA) was applied to probe sets that had a present detection call in at least 4 out of 6 CCD samples. Using *limma,* the average expression value for each experimental condition was estimated. Based on these estimates, the contrasts CTC vs. T, CTC vs. P, CTC vs. G, T vs. P, T vs. G, and P vs. G were estimated. A moderated t-statistic (implemented in *limma*) was used to test whether each contrast was significantly different from 0 Resulting p-values were corrected for multiple testing with Benjamini-Hochberg (BH) to control for false discovery rates [10]. Subsequently, a filter was applied selecting genes that were significantly 2-fold up– AND/OR downregulated using the BH corrected p-values (p < 0.05) in the comparisons CTC vs. T, AND in CTC vs. P, AND CTC vs. G. The resulting filtered data were functionally analyzed with the Ingenuity Pathway Analysis (IPA) application (Ingenuity® Systems, http://www.ingenuity.com). Gene networks were generated within the Ingenuity Pathway Analysis programme, and ordered by a score. This score is a numerical value used to rank networks according to their degree of relevance to the Network Eligible molecules in the dataset. The network score is based on hypergeometric distribution and is calculated with the Ingenuity Pathway Analysis programme using the right-tailed Fisher's Exact Test. The set of genes upregulated in CTC vs. T, AND in CTC vs. P, AND CTC vs. G was further explored for genes associated with cell motility using AmiGO, the GO Consortium’s annotation and ontology tool kit [11]. Gene expression data are publicly available online via: http://www.ncbi.nlm.nih.gov/geo/query/acc.cgi?token=zbotdukywssgujm&acc=GSE18670.

### Validation of gene signature

Between 2006 and 2010, tissue samples were obtained from patients who had undergone pancreatic resection for PDAC. Samples were stored at −80°C in RNA*later®* (Qiagen). To verify that the tumor samples contained more than 70% tumor, hematoxylin and eosin stains were made from each tumour sample and from surrounding control pancreatic tissue to confirm its non-cancerous histology. True cellularity was not determined. From the primary tumour of 143 patients and from surrounding non-tumoural pancreatic (control) tissue of 14 patients, total RNA was extracted using the RNeasy Mini kit according the manufacturer’s instructions. Only samples with an RNA integrity number (RIN) of ≥ 7.0 were used for further analysis, i.e. 78 PDAC-samples (M/F = 40/38, median age 64 y. (range 32–80 y.)) and 6 controls. A limited set of genes was selected based on the results from the microarray gene expression analysis in CTC. Using the nCounter system (Nanostring Technologies, Seattle, WA) gene expression levels were quantified at the VIB Nucleomics Core of the VIB Microarray Facility.

### Survival analysis

Median overall survival (OS) and disease-free survival (DFS) rates in our patient group were 18.7 months (95% CI: 12.4-25.6 months) and 10.0 months (95% CI: 7.4-12.4 months), respectively. Uni- and multivariable Cox regression analyses were performed to identify predictors of DFS and OS rates after pancretic resection with curative intent. The common closing date was the 1st of July 2011. Variables considered were dichotomized expression values of the genes of interest and clinico-pathological variables: tumour localisation in the pancreas (head/corpus/tail), tumour size (< 20mm, 20-40mm, > 40mm), tumour differentiation grade (pG), pT, pN, pM, extracapsular lymph node involvement (ECLNI), perineural invasion (PNI), lymphovascular invasion (LVI), vascular invasion (VI), resection margin status (R), minimal resection margin > 1mm, adjuvant chemotherapy within the first 3 months after surgery. The cut-off for dichotomisation of gene expression values was the median fold change calculated for every gene. All variables with p-values ≤ 0.10 in univariable analysis were considered for the final multivariable model. A p-value ≤ 0.05 was considered statistically significant.

## Results

### Spiking experiments

**Figure 3:**
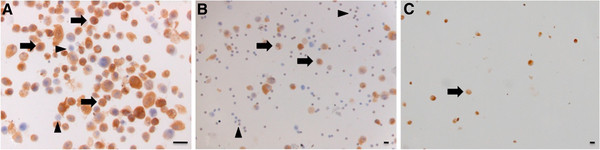
**A. Prekeratin staining of cytospins of sorted CTC fraction after spiking 5.0 × 10**^**6**^**Panc-1 cells in 20ml EDTA-treated whole blood.** Note the marked cytoplasmatic prekeratin staining of almost all sorted cells indicating very high purity of the sorted fraction. The stained cells have large blue nuclei with prominent nucleoli (arrow) indicating the malignant nature of the enriched PANC-1 cells. PBMCs are marked with an arrowhead. Magnification: 30x, Bars: 25μm. B - Prekeratin staining of cytospins of sorted ‘gated’ fraction (G) after spiking 5.0 × 10^6^ Panc-1 cells in 20ml EDTA-treated whole blood. The sample is mainly composed of two cell types: most are PBMCs with a little amount of non-staining cytoplasma (arrowheads) and a few larger cells with prominent cytoplasmatic prekeratin staining (Panc-1 cells) (arrows). Magnification: 10x, Bars: 25μm. 3C - Prekeratin staining of the sorted CTC fraction after spiking 5.0 × 10^4^ Panc-1 cells in 20ml EDTA-treated whole blood. Cell density is greatly reduced owing to the lower concentration of spiked Panc-1 cells. Despite varying cell size, marked cytoplasmatic prekeratin staining was seen in most cells, indicating high purity of the sorted cells. Magnification: 10x, Bars: 25μm.

Cytospins after FACsorting of the high Panc-1 concentration whole-blood samples showed marked cytoplasmatic prekeratin staining of >90% cells, indicating that this fraction was composed almost exclusively of epithelial cells. Omnipresent large nuclei with prominent nucleoli demonstrated the malignant nature of these cells, indicating that these were the spiked Panc-1 cells (Figure 3A). In contrast to the CTC-fraction, the G-fraction in both sortings was composed mainly of PMBCs (>75%), very few larger cells with prominent cytoplasmatic prekeratin staining (Panc-1 cells) and cellular debris (Figure 3B).

Similarly, 5.0 × 10^4^ Panc-1 cells (low concentration) were spiked in 20ml of EDTA-treated whole blood. The cytospins’ cell density was greatly reduced owing to the lower concentration of spiked Panc-1 cells and lower recovery of spiked cells. Despite varying cell size, marked cytoplasmatic prekeratin staining in the CTC-fraction could be demonstrated (Figure 3C). On the other hand, the G-fraction was composed almost exclusively of PBMCs similar to the G-fraction after sorting of the high Panc-1 concentration. Estimation of the recovery after spiking, density centrifugation, FACsorting and cytospins was not attempted.

### RNA purity and integrity

Similar to enrichment procedures used in our spiking experiments, blood of ten patients (Table 1) was sorted into two fractions (CTC and G). A median number of 1,828 (range: 761 – 33,337) presumed CTC was obtained for RNA extraction. A260/A280 ratios were > 1.80 for all samples. Tissue samples (T and P) were obtained with good RNA integrity numbers (RIN) ≥ 7.5, except for samples T123 and P138, which had a RIN of 7.1 and 6.9, respectively. The RNA quality standards set for microarray analysis were reached in 6 out of 10 patients for all 4 subgroup samples, i.e. for the CTC, G, T and P samples the median starting amount of RNA converted and amplified to antisense cRNA was 0.315ng (range: 0.22 – 0.49), 8.00ng (range: 0.528 – 10), 10ng (range: 5–10) and 10ng (range: 5 – 10), respectively.

### Gene expression analysis

For 46,467 probes, fewer than 4 out of 6 CTC samples gave present detection calls and therefore these probes were omitted from further analysis (Additional file 1). We retained a final set of 8,152 probes for statistical analysis. Differential gene expression was observed between CTC and T samples for 3,570 genes, of which 1,645 genes were significantly upregulated and 1,925 genes downregulated (Table 2). Between CTC and P samples a total of 3,669 genes were differentially expressed. Only 6 genes were found to be significantly upregulated in T vs. P samples, i.e. S100 calcium binding protein (S100P), tubulin α-4a (TUBA4A), interleukin 1 receptor antagonist (IL1RN), epithelial cell transforming sequence 2 oncogene (ECT2), stratifin (SFN), and chromosome 19 open reading frame 33 (C19orf33). No genes were found to be significantly downregulated in T vs. P samples.

**Table 2:** Counts of down- and upregulated genes

	**Corrected p-value < 0.05**	
	**Log-ratio < −1**	**Log-ratio > +1**
CTC vs T	1,925	1,645
CTC vs P	1,951	1,718
CTC vs G	825	905
T vs P	0	6
T vs G	1,139	1,089
P vs G	1,253	1,128

Log-ratio < −1 indicates significant 2-fold downregulation; Log-ratio > +1 indicates significant 2-fold upregulation.

### Molecular network and pathway analysis

To avoid probes referring to the same molecule in the IPA application, a maximum of fold-change values was taken. In order to compare CTC vs. T, AND CTC vs. P, AND CTC vs. G fractions, only genes with a significant 2-fold up– or downregulation were selected (corrected p-values < 0.05). This selection resulted in a total of 1,059 probe sets. Among these 1,059 differentially expressed genes, 738 molecules were eligible to generate gene networks with the Ingenuity Pathway Analysis software, and a total of 572 molecules were eligible for the generation of cellular functions and pathways. Primary functions involved in the top gene network were cancer, cell death, and neurological disease, with transforming growth factor β1 (TGF-β1) as the core molecule (Additional file 2) TGF-β1 was found to be highly upregulated in the CTC fraction compared to the T, P, and G fractions, with a fold change (FC) of 3.18, 3.33, and 2.16, respectively. The three most important biofunctions for the category ‘Diseases and Disorders’ were inflammatory response (6.6E-04 < p-value < 4.81E-02), cancer (1.69E-03 < p-value < 3.92E-02), and genetic disorders (1.69E-03 < p-value < 3.92E-02). The three most important ‘Molecular and Cellular functions’ were cell-to-cell signaling and interaction (7.84E-04 < p-value < 4.98E-02), cellular development (2.47E-03 < p-value < 4.81E-02), and cellular movement (5.22E-03 < p-value < 4.74E-02). P-values were calculated with the Ingenuity Pathway Analysis program using the right-tailed Fisher's Exact Test and represent a measure of its statistical significance with respect to the functions for the dataset and the references of molecules (which define the molecules that may have been functions eligible).

The pathway with the highest expression ratio (10.5%) was p38 mitogen-activated protein kinase (MAPK) signaling. This ratio was calculated as the number of molecules in a given pathway that met cut-off criteria (n=10) divided by the total number of molecules making up that pathway (n=95). In the p38 MAPK pathway TGF-β1, cytosolic phospholipase A2 (cPLA2), and MYC associated factor X (MAX) were significantly upregulated in the CTC fraction as compared to the T, P, and G fractions (p < 0.05). In addition, using the gene ontology application software AmiGO, the list of upregulated genes associated with both p38 MAPK signaling and cell motility was mapped. This resulted in a set of 9 genes, i.e. arachidonate 12-lipoxygenase (ALOX12), autocrine motility factor receptor (AMFR), Rho-guanine nucleotide exchange factor 2 (ARHGEF2), CCL5, engulfment and cell motility protein (ELMO1), signal transducer and activator of transcription 3 (STAT3), Talin-1 (TLN1), Tropomyosin alpha-4 chain (TPM4) and Vinculin (VCL).

### Clinically prognostic value of cell motility gene signature

**Figure 4:**
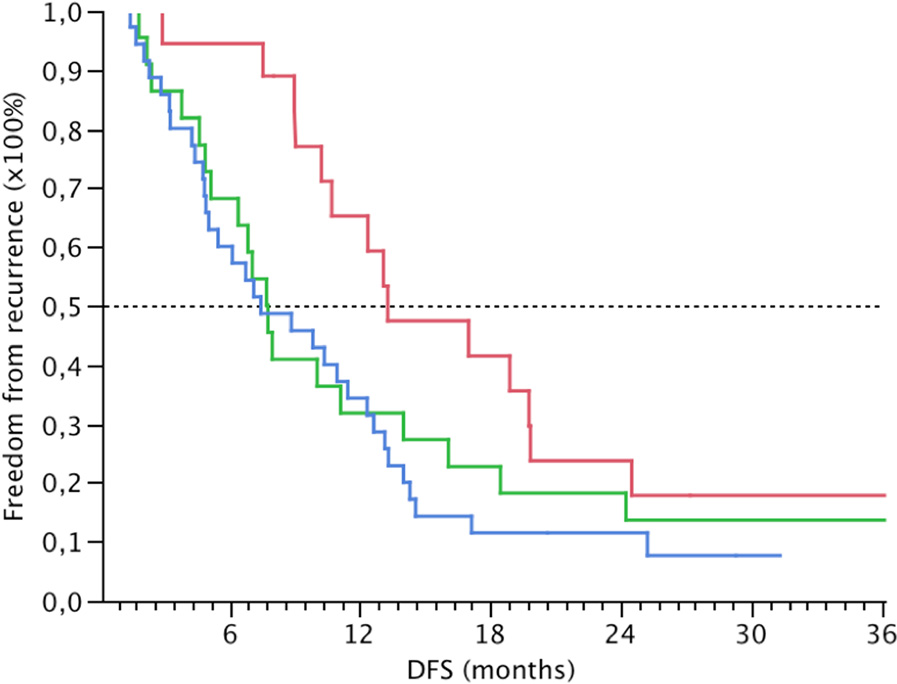
**Kaplan-Meier estimated disease-free survival for the validation set.** p-values for tests between groups were p=0.099 (Log-Rank) and p=0.028 (Wilcoxon). Grouping was done according to overexpression of ≥ 4 motility genes or/and TGF-β1. No overexpression of ≥ 4 motility genes or TGF-β1 = group 0 (red). Overexpression of ≥ 4 motility genes or TGF-β1 = group 1 (green). Overexpression of ≥ 4 motility genes and TGF-β1 = group 2 (blue).

**Figure 5:**
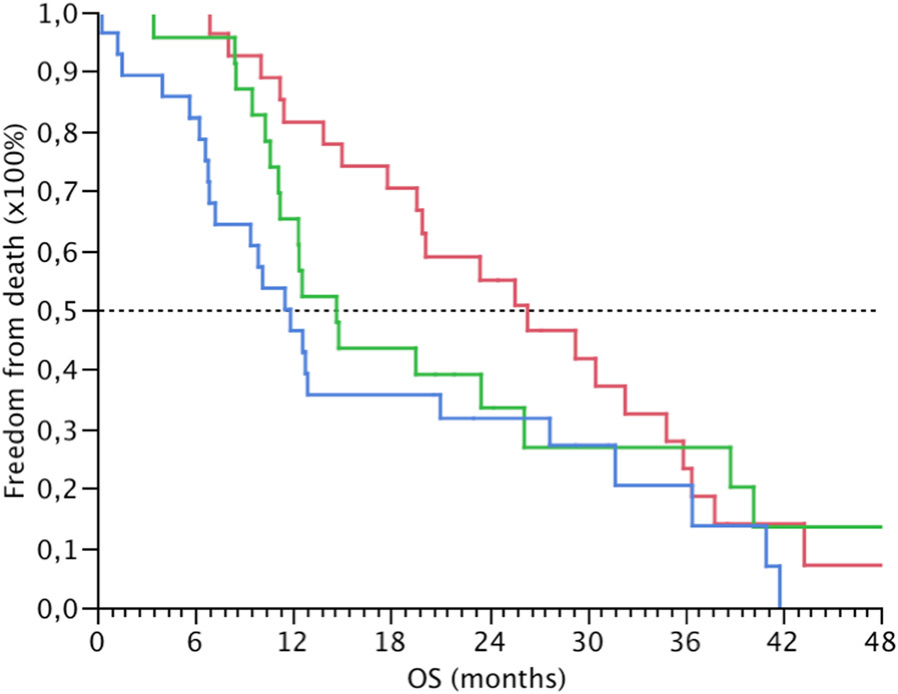
**Kaplan-Meier estimated overall survival for the validation set.** p-values for tests between groups were p=0.155 (Log-Rank) and p=0.018 (Wilcoxon). Grouping was done according to overexpression of ≥ 6 motility genes or/and TGF-β1. No overexpression of ≥ 6 motility genes or TGF-β1 = group 0 (red). Overexpression of ≥ 6 motility genes or TGF-β1 = group 1 (green). Overexpression of ≥ 6 motility genes and TGF-β1 = group 2 (blue).

Using the nCounter system, expression values of 12 genes (Table 3) obtained from the microarray data were studied in 78 PDAC samples, and represented as the median (range) fold change expression of tumour vs. controls. Univariable Cox regression analysis did not show any significant correlation between the continuous fold change values and survival. After dichotomization a significant correlation was found on univariable analysis for TGF-β1 and DFS (p=0.05, HR (95% CI) = 1.903 (1.053 – 3.530)). For OS, a correlation was found but it did not reach statistical significance (p=0.08, HR (95% CI) = 1.56 (0.94 – 2.62)). Next, a combined variable was created containing information on TGF-β1 and the number of motility genes expressed above the median in the validation set. When the fold change of ≥ 4 of the 9 motility genes and/or TGF-β1 was high, DFS was significantly worse on univariable analysis (p=0.033, HR (95% CI) = 1.84 (1.04 – 3.46)) (Table 4 and Figure 4). When the fold change of ≥ 6 of the 9 motility genes and/or TGF-β1 was high, worse OS was found (p=0.067, HR (95% CI) = 1.734 (0.962 – 3.132)) (Table 5 and Figure 5).

**Table 3:** Median (range) fold change PDAC/control tissue (nCounter) for the validation set

**Gene**	**Fold change**	
	**Median**	**Range**
TLN1	12.10	10.62 – 15.29
STAT3	12.10	10.90 – 13.07
VCL	11.59	9.99 – 12.56
CCL5	9.82	7.67 – 11.79
AMFR	10.73	9.73 – 11.54
TPM4	14.26	12.67 – 15.64
ALOX12	0 (0.44) *	0 – 6.45
ARHGEF2	10.21	8.44 – 10.97
ELMO1	7.66	5.53 – 9.00
TGF-β1	10.74	8.76 – 11.64
MAX	10.83	9.56 – 11.38
PLA2G4C	6.73	4.89 – 8.49

* between parenthesis is mean.

**Table 4:** Univariable and multivariable Cox regression analysis for disease-free survival

	**Univariable**		**Multivariable**	
	**p-value**	**HR (95% CI)**	**p-value**	**HR (95% CI)**
Tumor location	0.105	1.628 (0.898 – 2.822)	0.065	0.557 (0.310 – 1.038)
pG	0.046		0.031	
1		0.597 (0.229 – 1.848)		0.431 (0.158 – 1.372)
2		1.207 (0.522 – 3.504)		0.899 (0.377 – 2.658)
3		2.023 (1.155 – 3.724)		2.089 (1.184 – 3.872)
TNM stage	0.004	3.454 (1.543 – 6.979)	0.013	2.978 (1.313 – 6.123)
≥4 genes or/and TGF-β1	0.033	1.903 (1.053 – 3.530)	0.041	1.885 (1.025 – 3.559)

HR (95% CI) hazard ration with 95% confidence interval, pG tumor differentiation.

**Table 5:** Univariable and multivariable Cox regression analysis for overall survival

	**Univariable**		**Multivariable**	
	**p-value**	**HR (95% CI)**	**p-value**	**HR (95% CI)**
pG	0.053		0.293	
1		0.738 (0.232 - 3.267)		.718 (0.220 – 3.220)
2		1.508 (0.545 – 6.251)		1.191 (0.418 – 5.004)
3		2.042 (1.132 – 3.922)		1.658 (0.885 – 3.312)
TNM stage	0.007	2.986 (1.394 – 5.828)	0.015	2.715 (1.234 – 5.499)
ECLNI	0.016	1.974 (1.141 – 3.361)	0.032	1.870 (1.057 – 3.268)
PNI	0.071	1.986 (0.947 – 4.879)	0.222	1.680 (0.750 – 4.521)
≥ 6 genes or/and TGF-β1	0.067	1.734 (0.962 – 3.132)	0.047	1.366 (1.004 – 1.861)

HR (95% CI) hazard ration with 95% confidence interval, pG tumor differentiation, pT tumor stage, ECLNI extracapsular lymph node involvement, PNI perineural invasion.

On multivariable analysis, high co-expression of TGF-β1 and the cell motility panel (≥ 4 out of 9 genes for DFS and ≥ 6 out of 9 genes for OS) were identified as an independent predictor of both DFS (p=0.041, HR (95% CI) = 1.885 (1.025 – 3.559)) and OS (p=0.047, HR (95% CI) = 1.366 (1.004 – 1.861)) respectively (Table 4 and 5).

## Discussion

Identification of specific genetic and phenotypic changes occurring in CTC could result in a better understanding of the metastatic process and lead to effective therapeutic strategies [12]. To our knowledge, for the first time global gene expression profiles were generated from blood enriched for CTC by a negative depletion technique. Depletion of dead (7AAD staining) and non-epithelial cell types (such as leukocytes (CD45+) and other endothelial/leukocyte progenitor cells (CD34+) results in a more unbiased enrichment of CTC both in spiking experiments and *in vivo* compared to *a priori* positive selection using epithelium-specific antibodies.

An important issue in small-sample gene expression microarray analyses is the quality of the final RNA sample. Indeed, all methods requiring additional steps (e.g. tissue biopsies from resection specimens, density centrifugation, FACS) to acquire very small amounts of cells bear the risk of RNA degradation. Moreover, the pancreas is known for its high concentration in RNases, leading to quick RNA degradation. In an effort to minimize this effect, periods of warm ischemia between removal of the surgical specimen and sample processing were kept to a strict minimum. Also, tissue samples were immediately submerged into a RNA stabilizing agent (RNA*later*®) and care was taken to store these samples under optimal conditions. Despite these stringent precautions moderate degradation was observed in a substantial number of samples. Nevertheless, moderate RNA degradation probably disturb microarray results only modestly [13], and the final number of samples with high-quality RNA was sufficient to draw sound conclusions from the present study. Microarray analyses showed differential gene expression profiles between the CTC, T, P and G fractions. In contrast to other reports, we found only 6 genes (C19orf33, ECT2, IL1RN, S100P, SFN, TUBA4A) to be significantly upregulated in the primary PDAC (T) as compared to the non-tumoural pancreatic tissue (P) samples, whilst no genes were found to be downregulated. This finding can be explained by the fact that the ductal epithelial cells, of which PDAC originates, make up only a small proportion of the bulk surrounding pancreas or tumour. Indeed, expression profiles often predominantly reflect the proportion of cell types and obscure more meaningful data. Another explanation may be that we finally retained only 8,152 probe sets out of 46,467 probes initially tested, because Robust Multichip Average (RMA) was applied only to probe sets with a present detection call in at least 4 out of 6 CCD samples.

In our stringent comparison of the various cell fractions (CTC vs. T, AND CTC vs. P, AND CTC vs. G), the top molecular and cellular functions expressed in CTC were cell-to-cell signalling and interaction, cellular development, and cellular movement. The pathway with the highest expression ratio in CTC was p38 MAPK signalling, which is known to be involved in biological processes such as inflammation, apoptosis, and cell differentiation [14]. More recently, the p38 MAPK signalling cascade was also shown to play a pivotal role in cancer cell migration, and could be stimulated by S100A8 [14]. Moreover, potential secretion of TNFα, VEGF-A and TGF-β1 from primary tumours induced S100A8 and S100A9, which in turn stimulated the enrichment of other inflammatory chemo-attractants. These findings suggest that an increased p38 MAPK activity in CTC could occur in response to S100A8 produced not only in homing organs (such as lungs, liver) but also in CTC. Stimulation of p38 MAPK by S100A8 has been shown to induce formation of pseudopodia important to dynamic cell movement in the process of invasion and extravasation [15]. Therefore, we used the gene ontology application software AmiGO at other genes that were overexpressed in CTC involved in the same process and in p38 MAPK signalling. Other genes mediating many of the component processes of invasion include Talin-1 and ELMO1. Both these genes were found to be upregulated in our CTC. Besides these genes, we found another 7 genes, i.e. ALOX12, AMFR, ARHGEF2, CCL5, STAT3, TPM4, and VCL. These 9 genes were termed CTC motility panel for ease of formulation.

In the p38 MAPK pathway TGF-β1, cPLA2, and MAX were significantly upregulated in CTC. TGF-β1 regulates a spectrum of cellular events, including cell proliferation, differentiation, and migration. In addition to the canonical Smad pathway, TGF-β1 can also activate MAPK, phosphatidylinositol 3-kinase (PI3K)/Akt and small GTPases in a cell-specific manner. Activation of ERK or p38 MAPK is required for both TGF-beta-induced epithelial-mesenchymal transition (EMT) and cell migration [16]. Reversible and local activation of TGFβ/Smad-signalling in breast cancer cells has recently been shown to cause a switch from cohesive movement to single cells motility and to promote haematogenous metastasis [17].

A clear association has been described between the CCL5/CCR5 axis and the directional migration and invasion of human cancer cells. In vitro, this has been shown to involve specific upregulation of matrix-metalloproteinase-9 (MMP-9) [18]. STAT3 is a transcription factor that has been linked with metastasis in different tumourtypes e.g. lung, colon adenocarcinoma, and PDAC [19]. Besides increasing cell motility and invasion, STAT3 is also thought to upregulate the expression of anti-apoptotic and growth-promoting genes and to facilitate the colonisation of the liver by tumour cells [20,21]. Therefore, STAT3 could be another new target for prevention of metastasis formation by CTC and for the treatment of established metastasis. STAT3 inhibitors are already available and currently undergo testing in various animal cancer models [20].

Our study provides a valuable source for researchers to explore. Using a negative depletion technique we were capable of isolating CTC, without using a priori selection markers. We have identified several candidate genes in CTC from PDAC, which can be used for various purposes. First, a combination of these genes may distinguish between benign and malignant disease of the pancreas. This was beyond the scope of this study. Also, as these genes represent the molecular signature of CTC in peripheral blood they may be useful for disease monitoring, prediction of survival, and response to therapy. Therefore we determined the expression level of our cell motility gene signature in 78 independent PDAC samples using the nCounter system. This system uses digital technology based on direct multiplexed measurement of gene expression, and offers high levels of sensitivity and precision [22]. We found that the combination of expression of our CTC motility panel and TGF-β1 identified a subgroup of primary PDAC at high risk of early recurrence and worse overall survival. Some of these genes may also be interesting therapeutic targets and need further exploration.However, we should remain cautious as measurement of mRNA levels to infer events at the protein level can be misleading, as discordances may exist between mRNA levels and encoded protein levels.

## Conclusion

Pancreatic CTC isolated from blood samples using a FACS-based negative depletion method, express a cell motility gene signature. The expression of this newly defined cell motility gene signature in the primary tumour enables us to predict survival of patients who undergo surgical resection for pancreatic cancer. The expression of this gene signature may be used as a selection tool in patients who are candidates for surgery, not only in pancreatic but also in other solid organ malignancies.

## Abbreviations

PDAC: Pancreatic ductal adenocarcinoma; T: Pancreatic tumoural tissue; P: Benign surrounding pancreatic tissue; CTC: Circulating tumour cells; G: Haematological and non-viable background.

## Competing interests

The authors declare that they have no competing interests.

## Authors’ contributions

GS participated in the study design, carried out the molecular assays, gathered the raw data, performed the statistical analysis and drafted the manuscript. TR carried out the pathological examination. RVE participated in the design of the study, coordinated the running of microarrays and performed statistical analysis. VVD participated in the design of the study and helped with data gathering. BT conceived of the study, participated in its design and coordination and helped draft the manuscript. All authors read and approved the final manuscript.

## Pre-publication history

The pre-publication history for this paper can be accessed here:

http://www.biomedcentral.com/1471-2407/12/527/prepub

## Supplementary Material

Additional file 1 SimpleAffy QC graph. Click here for file

Additional file 2 Top functions and genes. Click here for file
