# Supplementary figures and images for: Pancreatic cancer circulating tumour cells express a cell motility gene signature that predicts survival after surgery
Source: BMC Cancer. 2012 Nov 16;12:527. doi: 10.1186/1471-2407-12-527 (PMC3599097; doi:10.1186/1471-2407-12-527)

SimpleAffy QC graph:


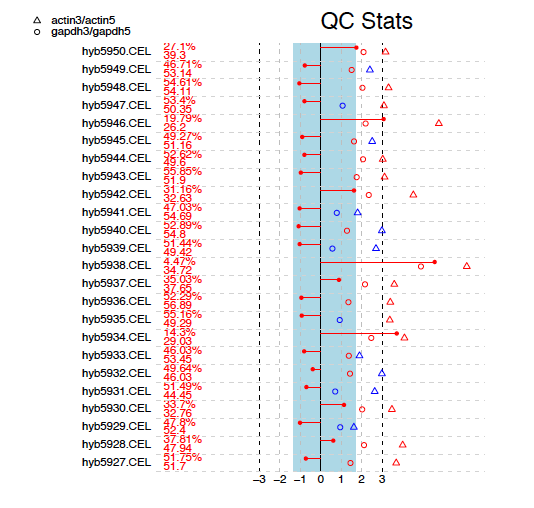


Hybridisation and Poly-A spike control plots:


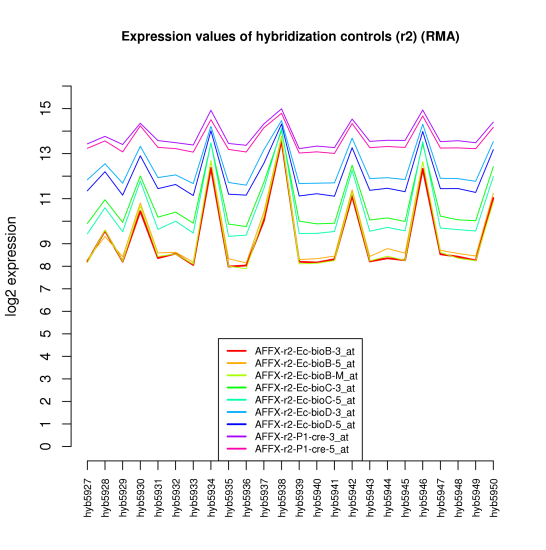

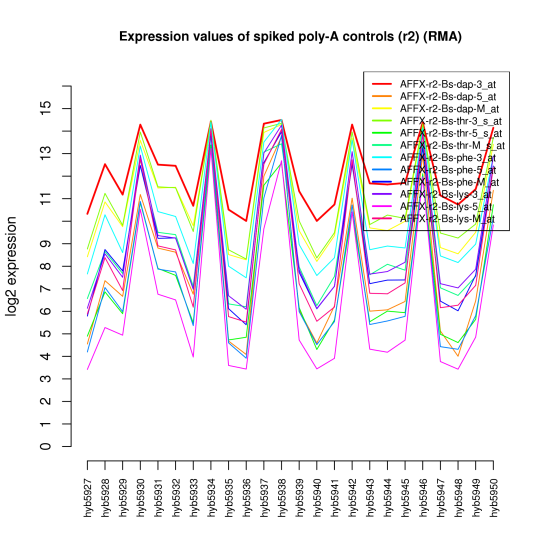

Supplement: Additional file 1 — SimpleAffy QC graph. [file 1471-2407-12-527-S1.docx]
