# Supplementary material for: Pancreatic cancer circulating tumour cells express a cell motility gene signature that predicts survival after surgery
Source: BMC Cancer. 2012 Nov 16;12:527. doi: 10.1186/1471-2407-12-527 (PMC3599097; doi:10.1186/1471-2407-12-527)
Supplement: Additional file 2 — Top functions and genes. [file 1471-2407-12-527-S2.docx]

| ID | Molecules in Network | Score | Focus Molecules (n) | Top Functions |
| --- | --- | --- | --- | --- |
| 1 | ADAP2, CAPRIN1, CARS, CCT2, CCT3, CCT4, CCT7, CCT6A, CNIH, DDIT4, DKC1, EEF1G, F13A1, FNDC3B, FNT1, G38P1, GCC2, GNPAT, HNMT, JMJD3, KIF3C, MTHFD2, MXD4, NET1, PD55A, Plasminogen activator, RAD21, RDHI1, SAR1A, SDPR, Smad2/3, SMC1A, STK17A, **TGFB1**, ZFP36L2 | 48 | 33 | Cancer, Cell death, Neurological disease |
| 2 | Akt, ATG12, BAT2, C1q, C1QBP, CD48, CR1, EIF3D, EIF3E, EPRS, GNAI3, GNAZ, GNGI1, GPSM3, HIST2H2AA3, IMMT, JTV1, KARS, LARS, NIPSNAP3A, PHIP, PPM1A, RBMX, RER1, RGS10, SCYE1, SELP, SERPING1, SF381, SFRS1, SFRS10, SFRS2IP, STK4, U2AF1, YTHDC1 | 48 | 33 | RNA Post-transcriptional modification, Cell-to-cell signaling and interaction, Hematological system development and function |
| 3 | ACTR6, AK2, B4GALT1, EEA1, EIF2AK1, GDI2, KLF3, MAP4K2, MARCH2, NAE1, NFkB, PJA2, PNKD, PPM1B, Rab5, RABA, RFTN1, RNF115, RUFY1, SEPT5, STX4, STX12, TAGLN2, TNFAIP2, TNFAIP8, TRAPPC9, UB13, UBE2, UBE2B, UBE2D2, UBE2E1, UBE2G1, UBE2H, USD1, WTAP | 46 | 32 | Post-translational modification, protein degradation, protein synthesis |
